# Validity and specificity of BOLD effects and their correction in ^1^H-fMRS

**DOI:** 10.3389/fnins.2024.1433468

**Published:** 2024-09-09

**Authors:** Nathalie Just

**Affiliations:** Danish Research Centre for Magnetic Resonance, Copenhagen University Hospital, Copenhagen, Denmark

**Keywords:** ^1^H-fMRS, rats, BOLD effects, metabolite concentrations, line broadening

## Abstract

**Purpose:**

This study aimed to characterize blood oxygen level-dependent (BOLD) effects in proton magnetic resonance (^1^H-MR) spectra obtained during optogenetic activation of the rat forelimb cortex to correct and estimate the accurate changes in metabolite concentration.

**Methods:**

For a more comprehensive understanding of BOLD effects detected with functional magnetic resonance spectroscopy (fMRS) and to optimize the correction method, a 1 Hz line-narrowing effect was simulated. Then, proton functional magnetic resonance spectroscopy (^1^H-fMRS) data acquired using stimulated echo acquisition mode (STEAM) at 9.4T in rats (*n* = 8) upon optogenetic stimulation of the primary somatosensory cortex were utilized. The data were analyzed using MATLAB routines and LCModel. Uncorrected and corrected ^1^H-MR spectra from the simulated and *in vivo* data were quantified and compared. BOLD-corrected difference spectra were also calculated and analyzed. Additionally, the effects of stimulated and non-stimulated water on the quantification of metabolite concentration swere investigated.

**Results:**

Significant mean increases in water and N-acetylaspartate (NAA) peak heights (+1.1% and +4.5%, respectively) were found to be accompanied by decreased linewidths (−0.5 Hz and −2.8%) upon optogenetic stimulation. These estimates were used for further defining an accurate line-broadening (lb) factor. The usage of a non-data-driven lb introduced false-positive errors in the metabolite concentration change estimates, thereby altering the specificity of the findings. The water and metabolite BOLD contributions were separated using different water scalings within LCModel.

**Conclusion:**

The linewidth-matching procedure using a precise lb factor remains the most effective approach for accurately quantifying small (±0.3 μmol/g) metabolic changes in ^1^H-fMRS studies. A simple and preliminary compartmentation of BOLD effects was proposed, but it will require validation.

## 1 Introduction

The investigation of neurochemical changes during brain activity is important for an improved interpretation of neurovascular coupling mechanisms in the healthy and diseased brains (Moreno et al., 2013; Logothetis et al., 2001). In this context, proton functional magnetic resonance spectroscopy (^1^H-fMRS) represents a technique of particular interest for investigating the foundations of functional MR imaging signals (Moreno et al., 2013; Logothetis et al., 2001) both in the human brain and animal models. ^1^H-fMRS techniques generate increasing interest for their ability to reproducibly assess activity-induced changes concentrations in key neurotransmitters, such as glutamate (Glu) and g-aminobutyric acid (GABA), and energy metabolites, such as glucose (Glc) and lactate (Lac) (Moreno et al., 2013; Mangia et al., 2009). Interestingly, absolute functional changes in these metabolite concentrations were found to be rather small (~0.2 μmol/g) in humans (Mangia et al., 2007a,b; Schaller et al., 2013). The absolute quantification of these changes was performed following the correction of ${\text{T}}_{2}^{*}$-induced effects (Zhu and Chen, 2001).

${\text{T}}_{2}^{*}$-induced effects [or blood oxygen level-dependent (BOLD) effects] induce the narrowing of spectral peak linewidths and T_2_ changes as a result of increased oxygenation within the activated region of interest. These decreases in the linewidth are mainly observed on water, N-acetylaspartate (NAA), and total creatine (tCr = PCr+ Cr) singlets and represent only 2% of the change during the visual stimulation of the human visual cortex (Schrantee et al., 2023; Ip et al., 2017). They are more easily observable as relative increases in a metabolite peak height of approximately 3% (Zhu and Chen, 2001). Although the change in metabolite concentration due to ${\text{T}}_{2}^{*}$-induced effects is low (< 1%), if left uncorrected, it may lead to a high degree of false-discovery rate (Bednarík et al., 2018). Errors may become particularly crucial when absolute changes as low as 0.2 μmol/g are expected. For an accurate estimation of metabolite concentration changes, Mangia et al. (2007a,b) proposed correcting for BOLD effects by line broadening the population-averaged stimulated spectrum to match the linewidth of the corresponding population-averaged REST spectrum. The corrected stimulated and REST spectra were subsequently subtracted, which resulted in a BOLD-free difference spectrum. Positive Glu and Lac peaks were visually identified and subsequently quantified using a simulated difference basis set within LCModel (Provencher, 1993). This procedure demonstrated a reproducible outcome within the human primary visual cortex at a high magnetic field owing to the large Signal to noise ratio (SNR) available (Mangia et al., 2007a,b; Schaller et al., 2013; Bednarík et al., 2018; Schaller et al., 2014; Lin et al., 2012; Bednarík et al., 2015).

In rodents, conducting ^1^H-fMRS studies remains challenging and has shown lower quantitative reproducibility (Just et al., 2013; Just and Sonnay, 2017; Xu et al., 2005). The methodology for obtaining accurate estimates of metabolite concentration changes during brain activation remains difficult to replicate in animal models with a voxel of interest (VOI) more than 500 times smaller than in the human brain (Just et al., 2013; Just and Sonnay, 2017; Xu et al., 2005; Just and Faber, 2019). The advent of revolutionary techniques such as optogenetics and chemogenetics that can be coupled with ^1^H-fMRS (Just and Faber, 2019) for the specific stimulation of excitatory cell populations could increase its potential if accurate quantification of metabolic concentration changes can be achieved.

While the difference spectrum procedure is amplitude-based and relatively straightforward, the line-broadening procedure followed by LCModel quantification is ambiguous because it involves calculating the areas underspectral peaks. On that account, peak line broadening should not affect metabolite quantification. However, this is not the case. Such errors in metabolite fitting models using linear combination modeling algorithms (such as LCModel) have been scarcely reported in the literature; nonetheless, there is a growing interest in improving the accuracy of metabolite concentration estimates and increasing the standardization across processing methods (Hong et al., 2019; Zöllner et al., 2021). In addition, these algorithms do not account for the change in the apparent T_2_ value.

In ^1^H-fMRS studies conducted at high magnetic field strengths, it is of paramount importance to remove BOLD effects or at least to determine how they affect neurochemical profiles. In human studies conducted at 7T with high SNR levels, corrections were applied and found to be adequate as their results aligned well with outcomes from difference spectrum procedures (Bednarík et al., 2018). How were these corrections optimized? How was it decided that they were suitable and how can they be appropriately applied to data with poorer SNR?

In the present study, BOLD effects and their correction were examined using simulated data and *in vivo* proton MR spectra acquired in rats during optogenetic stimulation of the primary somatosensory forelimb cortex (S1FL). This preliminary research aimed to investigate the robustness of the line-matching procedure to correct for BOLD effects and to determine the validity of this correction within LCModel. In addition, a simple method to better isolate and separate BOLD contributions from metabolite concentrations was proposed.

## 2 Methods

### 2.1 Simulations

A highly resolved proton magnetic resonance (^1^H-MR) spectrum acquired at 9.4T with a stimulated echo acquisition mode (STEAM) sequence and a mouse cryoprobe (Bruker Biospin GmbH, Ettlingen, Germany) in the thalamus of a healthy mouse was used to represent a stimulated ^1^H-MR spectrum (STIM). This ^1^H-MR spectrum was line-broadened (lb = 1 Hz) so that the difference in the spectral amplitude of NAA represented a 2% change according to values presented in the literature and represented the REST ^1^H-MR spectrum (REST). White noise was added to compensate for the smoothing effect. The difference in the NAA amplitude was assumed to represent the line-narrowing effect induced by the BOLD effects. Metabolite concentrations were quantified using LCModel, which used two reference standards: (a) the internal water signal from the unsuppressed water scan acquired in the same mouse, which was assumed to represent the BOLD contaminated water signal (for clarity purpose, it was called waterBOLD) and (b) the internal water signal from the unsuppressed water scan acquired in the same mouse and line-broadened (to represent a 2% signal decrease compared to waterBOLD), which was assumed to represent the water signal at REST (waterREST).

### 2.2 Animals and surgery

All experiments were performed according to the German Tierschutzgesetz and were approved by local authorities (Landesamt für Natur, Umwelt und Verbraucherschutz Nordrhein-Westfalen, Germany). A total of eight female Fisher rats (F344) were included in the present study. Each of them underwent two craniotomies above the primary somatosensory forelimb (S1FL) cortex for the injection of a viral construct (Yizhar et al., 2011) and the optic fiber (OF) implantation on the day of MR acquisitions. An OF of 200 μm in diameter was inserted only superficially above the S1FL at a depth of 100 μm and glued to the skull. Each rat was intubated under isoflurane anesthesia in oxygen (2–2.5%), and one of the caudal veins was catheterized for infusing pancuronium bromide (1 mg/ml BW) during functional magnetic resonance imaging (fMRI)-fMRS acquisitions. The rat skull was covered with warm agarose gel (1%) to further prevent air-tissue susceptibility artifacts. After fixing the rat head using ear and bite bars, the rat was positioned in a dedicated cradle. Respiration was monitored throughout the entire duration of the experiments, and end-tidal CO_2_ measurements were performed using a capnometer (CapStar-100 CO_2_ Analyzer, CWE Inc., Ardmore, PA, USA) and maintained between 2.8% and 3.5%. The temperature was measured through a rectal probe and was maintained at 37 ± 1°C via water tubing linked to a temperature retro-controlled bath.

### 2.3 Magnetic resonance Imaging and spectroscopic experiments

#### 2.3.1 BOLD-fMRI

All MR experiments were performed at 9.4T (Biospec 94/20, Bruker Biospin GmbH, Ettlingen, Germany) in a small animal MR scanner equipped with 0.7 m/T gradients using a 20 mm-single loop surface coil for reception and a 90-mm volume coil for transmission (Rapid Biomedical gmbH, Rimpar, Germany). After the acquisition of pilot and anatomical images, anesthesia was switched to medetomidine (Domitor, Pfizer, Orion Corporation, Espoo, Finland) [0.04 mg/kg (bolus) + 0.05 mg/kg/hr (subcutaneous infusion)] and isoflurane was discontinued. The first single-shot gradient-echo echo-planar imaging (TR/TE= 1,000/18 ms; FOV = 28 × 26 mm; Matrix = 80 × 80; Bandwidth = 200–300 kHz; TH = 0.8 mm; 16 slices; 600 images) took place at least 1 h after the start of the medetomidine infusion and after the whole-brain shimming using MAPSHIM. Laser pulses were delivered successively using in-house developed programs, which allowed triggering and repetition of a 10 s OFF-10 s ON-10 s OFF paradigm (pulse frequency of 9 Hz; pulse duration of 10 ms). A power-calibrated green laser light (552 nm) was delivered, ensuring a mean power intensity below 22 mW/mm^2^ at the tip of the OF to avoid heat effects (Christie et al., 2013). BOLD-fMRI images were processed using SPM12 as described earlier (Just et al., 2013).

#### 2.3.2 Functional magnetic resonance spectroscopy

Localized functional proton MR spectroscopy was conducted using a STEAM sequence (TR = 4 s; TE = 2.6 ms; TM = 10 ms SW = 4,960 Hz; 4,096 points). An 8 μl voxel of interest (VOI) was positioned onto the anatomical T_2_-weighted RARE images co-registered to the GRE-EPI images, ensuring that most of the VOI encompassed the BOLD responses, while lipid contamination from the skull was minimized. The water signal was suppressed using the VAPOR module (Tkác et al., 1999), and three modules of outer volume saturation (OVS) were interleaved with the water suppression pulses. A 3 × 3 × 3 mm^3^ voxel was placed over the rat cortex and used for shimming down to a water linewidth of 15 Hz, using first and second-order FASTMAP shimming. A 2.5 min OFF-5 min ON-2.5 min OFF paradigm was used, representing 45 min of acquisition [650 Free Induction Decays (FIDs)] and repeated 4 times per rat. Unsuppressed water spectra were also acquired using the same sequence and paradigm to provide reference water peaks for eddy current correction and further metabolite quantification. STEAM ^1^H-MR spectra were reconstructed and preprocessed using in-house written MATLAB routines. For each rat, raw ^1^H spectra were corrected for frequency drift and FIDs were summed across stimulation and rest periods and transferred to a SUN station for the LCModel analysis (Provencher, 1993) using a basis set provided by Steven Provencher, which contained a simulated set of macromolecules and the following metabolites: Scyllo-Inositol: Scyllo; Alanine: Ala; Aspartate: Asp; Glycero-phosphocholine: GPC; Phosphocholine: PCh; Creatine: Cr; Phosphocreatine: PCr; γ-aminobutyric acid: GABA; Glucose: Glc; Glutamine: Gln; Glutamate: Glu; Glutathione: GSH; myo-inositol: Ins; Lactate: Lac; N-acetylaspartate: NAA; N-acetylaspartyl-glutamic acid: NAAG; Phosphatidylethanolamine: PE; Taurine: tau; tCr: total creatine (PCr + Cr); and Glx: Glu + Gln. Absolute metabolite concentrations were obtained using unsuppressed water signals as an internal reference, assuming a brain water content of 80%. The Cramer–Rao lower bounds (CRLBs) were used as a reliability measure of the metabolite concentration estimates. Only metabolites with CRLBs below 15% were retained for further analysis.

### 2.4 Data analysis

#### 2.4.1 Estimation of ${\text{T}}_{2}^{*}$-induced effects on NAA and tCr spectral peaks

A moving average was used to calculate the NAA and tCr signal time courses. Each metabolite concentration time point was obtained by summing four consecutive blocks of eight FIDs. The procedure was applied to each of the four functional MR spectroscopic acquisitions obtained during the delivery of the green laser paradigms. The time courses were then averaged across the animal population. The temporal resolution was 42 s. Unsuppressed water signal time courses were obtained using a 4 s temporal resolution for each rat and averaged over the population of rats.

#### 2.4.2 Estimation of metabolite concentration changes

After phasing and correcting for B0 shifts, 1,200 FIDs were summed per animal and then aggregated across 8 rats for the 5-min rest periods and 5-min stimulation periods, which resulted in a STIM spectrum and a REST spectrum. The STIM and REST spectra were then individually fitted using LCModel. Metabolite concentrations were obtained using either the temporally averaged unsuppressed water peak obtained during the resting periods or the temporally averaged unsuppressed water peak obtained during the stimulation periods. The latter procedure was termed as the waterBOLD approach, as described in the theory section.

The STIM and REST spectra were also subtracted from each other. BOLD-corrected STIM spectra (STIMC) were obtained using a line-broadening factor lb. Furthermore, lbs were increased in steps of 0.2 Hz and applied to the NAA peak of the averaged stimulated spectrum to match the linewidth of the NAA peak of the averaged REST spectrum. The lb value that best minimized the residual NAA difference peak between STIM and REST was used. The STIMC spectra were fitted using LCModel.

All procedures were performed using an in-house written MATLAB routine. BOLD-corrected difference spectra (STIMC-REST) were also obtained and fitted using LCModel after the simulation of a basis set containing positive lactate and glutamate changes and negative aspartate and glucose changes.

### 2.5 Statistics

A Shapiro–Wilk test was performed for all the metabolite concentrations quantified during REST and stimulation conditions, demonstrating normality of all the distributions with a *p*-value above 0.05. Repeated measures one-way ANOVA tests followed by Bonferroni *post-hoc* tests (11 metabolites) to correct for multiple comparisons were employed to compare the changes in the metabolite concentrations. The results were presented as mean ± CRLB. A *p*-value under 0.05 was considered significant.

The averaged percent changes in the signal time courses between the REST and stimulation periods were compared using a paired Student's *t*-test. A *p*-value under 0.05 was considered significant.

The results were presented as mean ± standard deviation.

## 3 Results

### 3.1 Simulations of BOLD responses obtained using fMRI and fMRS

#### 3.1.1 Comparison of BOLD responses

For a better understanding of the relationship between ${\text{T}}_{2}^{*}$ and spectral peak linewidths during localized brain activation, equations relating to these entities were used.

The full width at half maximum (FWHM) of a spectral peak obeys the following equation:


(1) $$\begin{array}{l} FWHM=1/\pi T_{2}^{*} \end{array}$$

Changes in ${\text{R}}_{2}^{*}$ due to focal cerebral activity can be expressed as follows:


(2) $$\begin{array}{l} \text{Δ}R_{2}^{*}=-1/T_{\text{E}}\times ln\left(1+BOLD\right) \end{array}$$

Allowing the change in the linewidth (Δlw) caused by BOLD effects can be expressed as follows:


(3) $$\begin{array}{l} \text{Δ}FWHM=1/\pi \text{Δ}T_{2}^{*}=-1/\pi T_{\text{E}}\times ln\left(1+BOLD\right) \end{array}$$

These equations were used to model and compare “BOLD-fMRI” and “BOLD-fMRS” responses on water.

As the magnetic field strength increases, the ${\text{T}}_{2}^{*}$ relaxation time decreases significantly (Peters et al., 2007). Upon the BOLD activation of the human motor cortex, changes in the relaxation rate Δ${\text{R}}_{2}^{*}$ increased linearly as a function of field strength (van der Zwaag et al., 2009). A higher spectral peak linewidth change due to activation should, therefore, be expected at a higher magnetic field strength for a same TE. The assessment of ${\text{T}}_{2}^{*}$-induced effects due to hyperoxygenation of blood should be easier at a high magnetic field strength (>7T).

**Figure 1:**
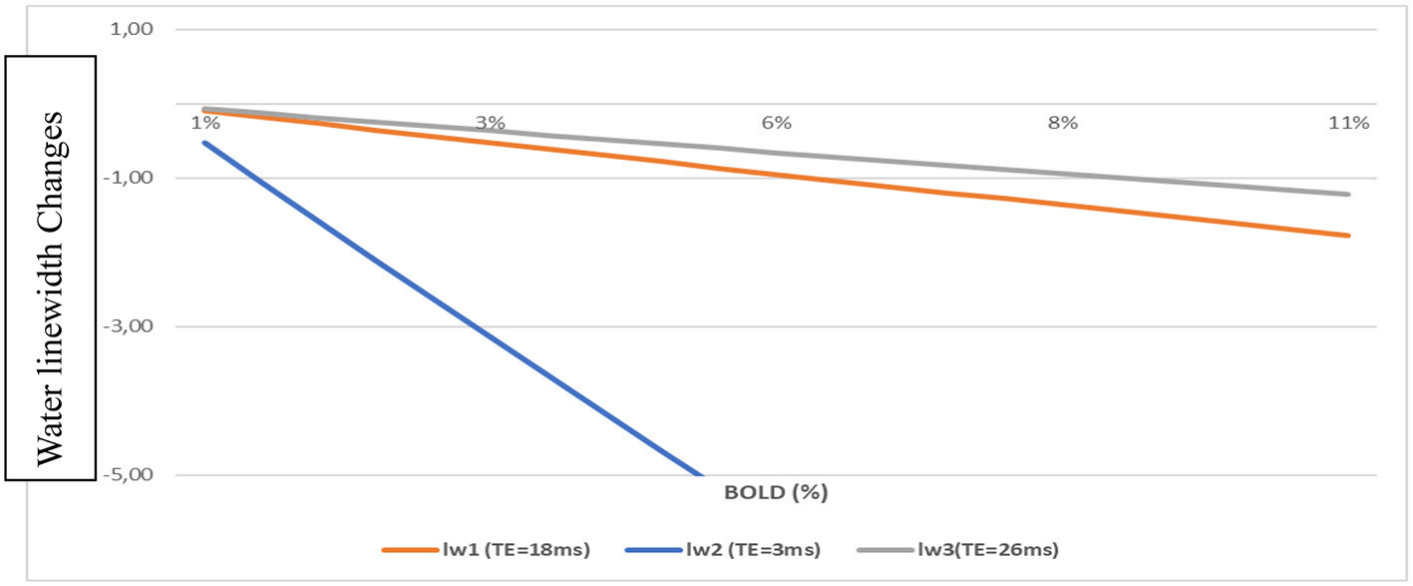
Water linewidth changes vs. BOLD-fMRI for different TEs using Equations (1) and (3).

Water linewidth changes (Δlw) for TE = 3, 18, and 26 ms, chosen from previous ^1^H-fMRS studies, were calculated with Equation (3) (Figure 1). The Δlw changes evolved linearly with the BOLD changes. At TE = 3 ms, the changes were elevated compared to those obtained at TE = 18 ms (9.4T, Just and Faber, 2019) or TE = 26 ms (7T, Mangia et al., 2007a,b).

For BOLD = 2% Δlw = −2.1 Hz (TE = 3 ms); Δlw = −0.35 Hz (TE = 18 ms); and Δlw = −0.24 Hz (TE = 26 ms).

However, for TE = 3 ms, the BOLD effects measured with fMRI were negligible. For Δ${\text{T}}_{2}^{*}$ = 1.5 s (arbitrarily chosen), BOLD = 0.47% for TE= 3 ms when BOLD = 2.9 % for TE = 18 ms. This change corresponded to a linewidth change of −0.5 Hz and BOLD = 0.5% for TE = 3 ms and BOLD = 3% for TE = 18 ms. These values well corresponded to linewidth changes quantified *in vivo* on NAA and Cr spectral lines (Schaller et al., 2014; Moussawi et al., 2011).

#### 3.1.2 Quantification of metabolite concentrations affected by simulated BOLD effects

**Figure 2:**
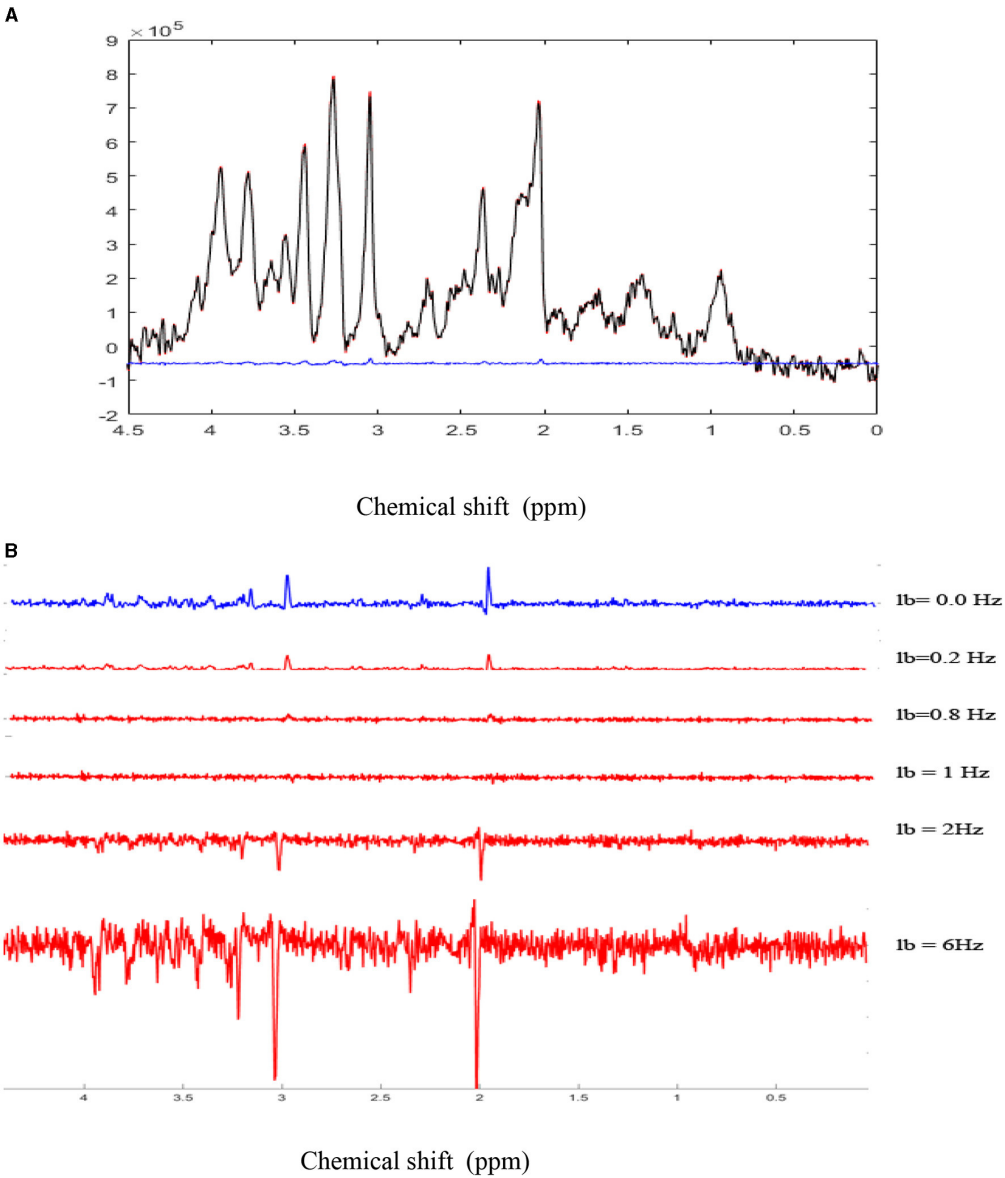
Simulation of BOLD effects: **(A)**. A highly spectrally resolved ^1^H-MR spectrum acquired at 9.4T in the mouse thalamus with a cryoprobe (red) served as a stimulated spectrum (STIM) and was exponentially line broadened by 1 Hz to represent a 2% amplitude decrease of the NAA peak in the resting spectrum (REST, black). The 2% difference represents the simulated BOLD effect as a result of ${\text{T}}_{2}^{*}$-induced effects. BOLD effects can also be seen as residual positive peaks in the difference spectrum (blue; STIM-REST) **(B)**. The REST spectrum was subtracted from the STIM spectrum, demonstrating residual positive peaks due to the simulated BOLD effect. As the line-broadening factor lb applied to the STIM spectrum increased from 0.2 Hz to 1 Hz, BOLD residuals gradually disappeared, while negative residuals increased for lb values above 1 Hz.

For illustrating errors induced by the BOLD effects and an erroneous correction of this effect on metabolite quantification, a 2% BOLD effect was simulated, which induced a 1-Hz decrease in the NAA spectral peak linewidth (Figure 2). The metabolite concentrations were quantified using LCModel (Provencher, 1993). In this example, no metabolic concentration changes occurred. Ground truth metabolic concentrations were obtained by the LCModel adjustment of the non-stimulated spectrum (black spectrum).

The correction for BOLD effects consists of the application of a line-broadening factor, which must be at least equal to the decrease of the spectral linewidth induced by focal cerebral activity so that stimulated and non-stimulated spectral linewidths are equivalent. This correction can be applied to the water peak (Zhu and Chen, 2001), but most ^1^H-fMRS studies have reported it on N-acetylaspartate (NAA) and total creatine (PCr + Cr) peaks (Mangia et al., 2007a,b; Schaller et al., 2013; Bednarík et al., 2018; Just et al., 2013). The MR spectra obtained during stimulation and REST were subtracted to observe changes induced by the BOLD effects. The residuals were visually observed and corresponded to the BOLD effects (Figure 2).

**Figure 3:**
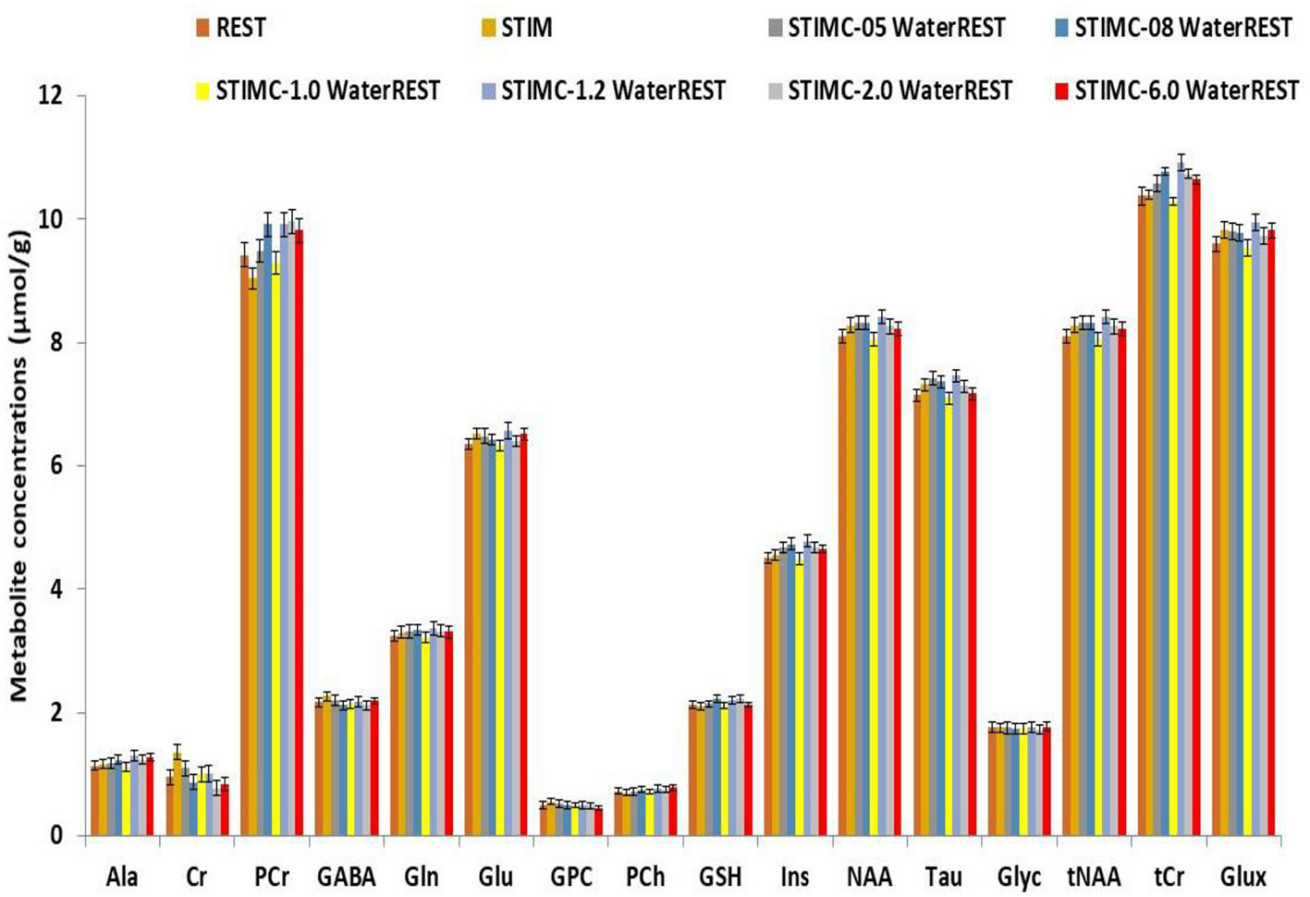
Comparison of neurochemical profiles. REST represents the ground truth for metabolic concentrations. Line-broadening (lb) factors of 0.5 Hz, 0.8 Hz, 1 Hz, 1.2 Hz, 2 Hz, and 6 Hz were applied to the simulated STIM spectrum. The correction that best approached the ground truth was for lb = 1 Hz.

The corrected neurochemical profiles of the spectra depicted in Figure 2 are displayed in Figure 3. REST represents the neurochemical profile for ≪true≫ metabolite concentrations, while STIMC represents the corrected neurochemical profile for which a 1 Hz line-broadening correction was applied. This correction represents the optimum correction for BOLD effects because the metabolite concentration differences were minimized. The line-broadening correction is the most effective when it is the closest to the linewidth reduction induced by BOLD effects. The error relative to REST remained below 1% for lb = 1 Hz for all the metabolites. The error rose to 4% for lb = 6 Hz for PCr and Ins and was of the same order of magnitude as the error induced by the non-corrected BOLD effects (lb = 0 Hz).

**Figure 4:**
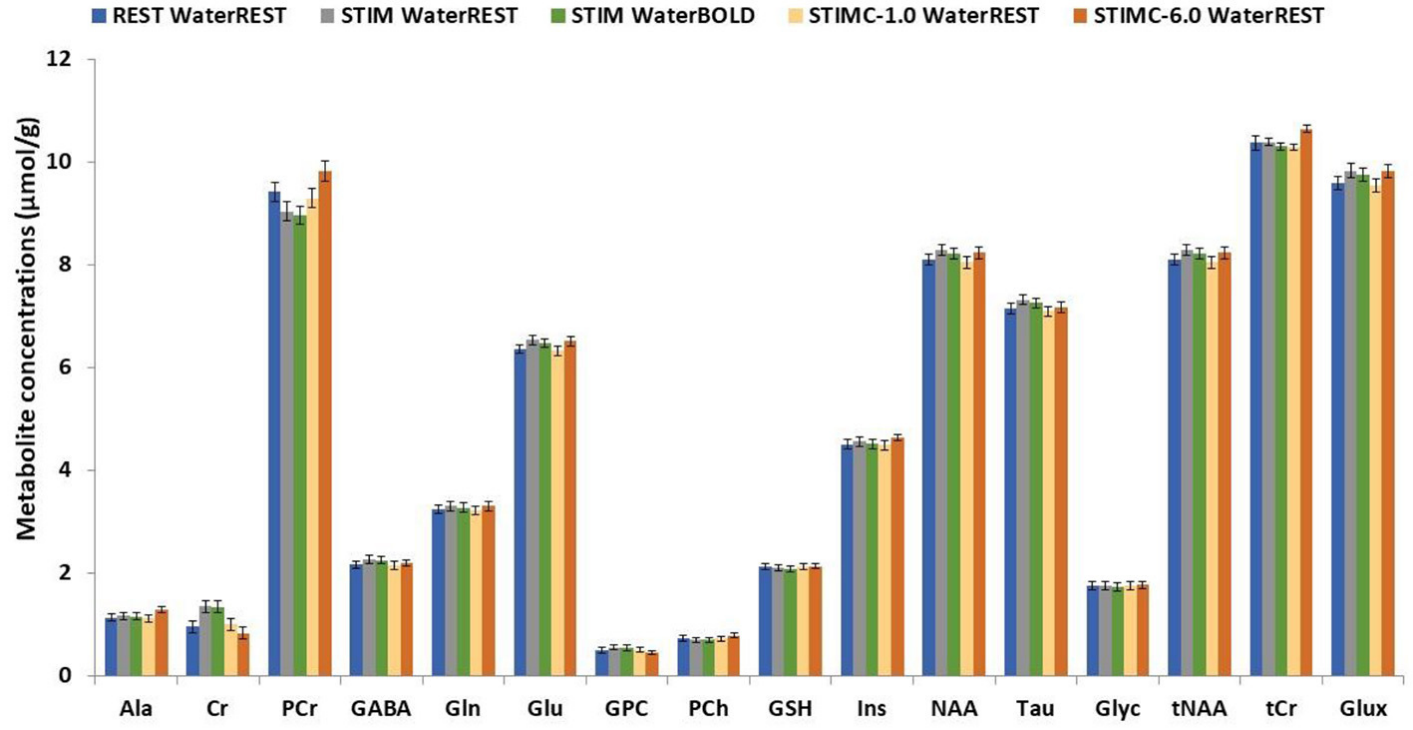
Comparison between the REST, STIM, STIMC, and waterBOLD neurochemical profiles. STIMC is the neurochemical profile of the line-broadened STIM spectrum with lb = 1 Hz. waterBOLD is the neurochemical profile of the STIM spectrum scaled with the stimulated water peak.

In Figure 4, STIM represents the uncorrected BOLD effect neurochemical profile. The quantification was performed with waterREST, the unsuppressed water spectrum acquired before the REST spectrum. BOLD effects increased the Glu concentrations by 2.6% and the NAA concentrations by 2.2%. “waterBOLD” represents the neurochemical profile acquired during stimulation but quantified using the simulated unsuppressed water peak with BOLD effects. Table 1 compares the Glu, NAA, and tCr concentrations quantified with LCModel for the different cases. When using waterBOLD for quantification, the BOLD effects increased the Glu concentrations by 1.8% and the NAA concentrations by 1.4%. The error induced by the BOLD effects, BOLD_water_, on the water was calculated as the metabolite concentration difference between STIM and waterBOLD and was approximately 0.8% for all metabolites. The error induced by the BOLD effects, BOLD_metab_, on the water was calculated as the metabolite concentration difference between STIM and STIMC. Interestingly, the BOLD effect-induced errors were cumulative: BOLD_STIM_ = BOLD_metab_ + BOLD_water_. Therefore, it is postulated that the estimates of metabolite concentrations with waterBOLD are affected by BOLD on metabolites only.

**Table 1:** LCModel quantification of Glu, NAA, and tCr (± CRLB) for the simulated REST and STIM spectra and STIM scaled with waterBOLD and the line-broadened STIM spectrum (STIMC lb = 1 Hz).

	**REST**	**STIM**	**waterBOLD**	**STIMC**
Glu (μmol/g)	6.36 ± 0.1	6.5 ± 0.1	6.48 ± 0.1	6.32 ± 0.1
NAA (μmol/g)	8.11 ± 0.1	8.3 ± 0.1	8.22 ± 0.1	8.05 ± 0.1
tCr(μmol/g)	10.38 ± 0.1	10.4 ± 0.1	10.31 ± 0.1	10.29 ± 0.1

### 3.2 *In vivo* characterization of BOLD effects

**Figure 5:**
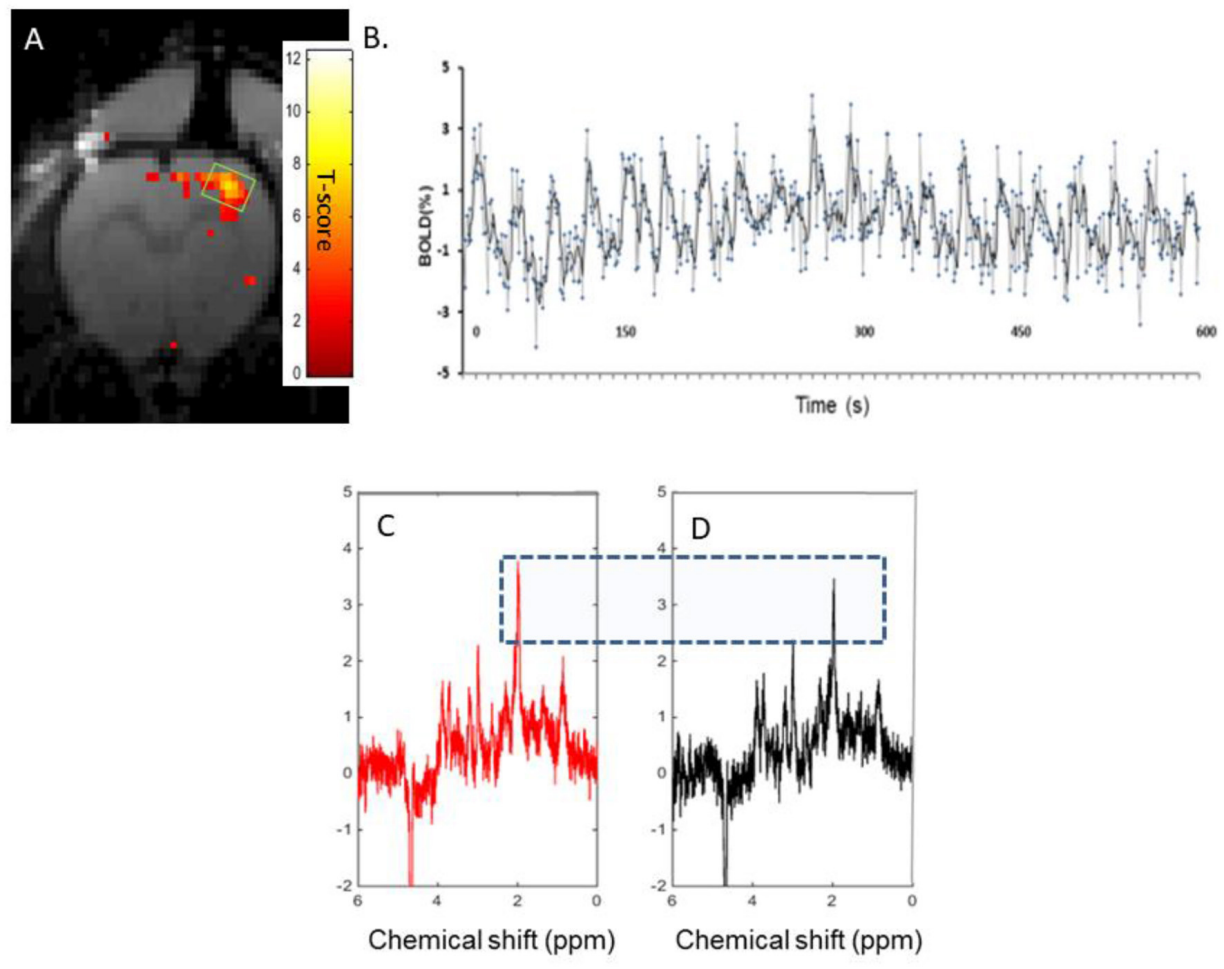
Characterization of BOLD effects and tCr signals: **(A)**. A typical T-value BOLD map overlaid onto an anatomical image of the rat brain, demonstrating activation in the S1FL in a rat upon optogenetic stimulation. **(B)** A typical BOLD time course during a 10s ON-20s OFF paradigm of stimulation lasting 10 min. C and D. Single rat ^1^H-MR spectra acquired in an 8 μl voxel of interest covering the activated NAA area (as shown in Figure 5A) during optogenetic stimulation (325 FIDs) [**(C)**, red] and resting periods [**(D)**, black]. Both spectra were reconstructed from FIDs acquired during the same paradigm of stimulation (5 min ON-5 min OFF, 45 min). The blue-dotted rectangle serves to show the slightly increased amplitude of NAA in the stimulated spectrum compared to the resting spectrum.

In the rat S1FL, optogenetic stimulation resulted in an average BOLD response measured using fMRI of 2.4 ± 1.3 % (Figure 5A). A typical T-value BOLD map overlaid onto an anatomical image is shown (Figure 5A), as well as a typical BOLD time course following a 10-min (10s ON-20s OFF) stimulation paradigm (Figure 5B). The representative raw ^1^H-MR stimulated (Figure 5C) and REST (Figure 5D) spectra obtained during the same paradigm of stimulation were acquired. The mean relative linewidth changes in NAA (Δlw_NAA_) were −2.8 ± 4 % (*n* = 8), and the mean relative tCr linewidth changes (Δlw_tCr_) were −1.4 ± 4% (*n* = 8).

**Figure 6:**
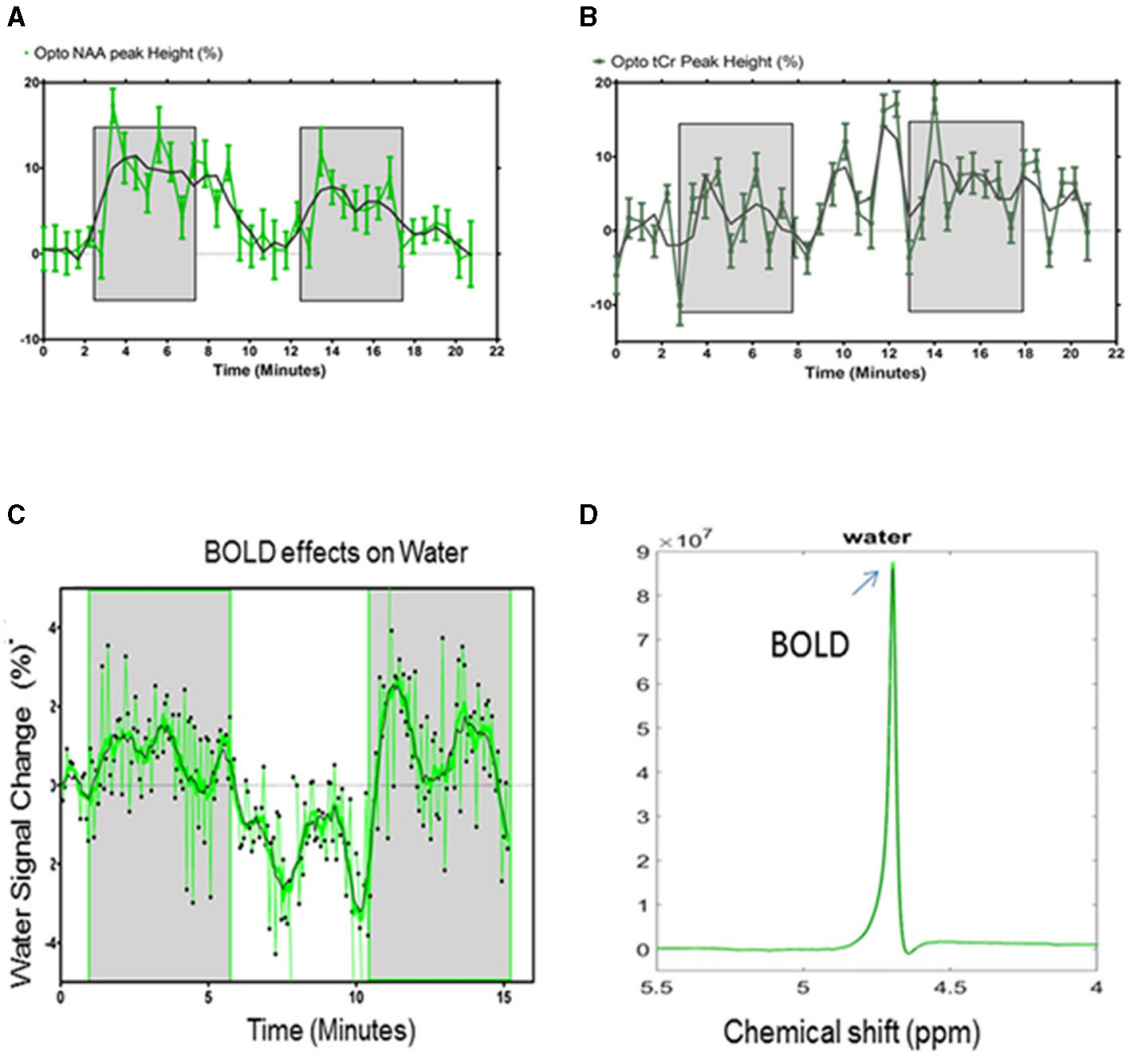
**(A)** Quantification of the NAA and **(B)** tCr relative peak height changes (% ± s.d) for the optogenetic stimulation. The shaded areas indicate the periods of stimulation. Statistical comparisons are described in the text. **(C)** Quantification of the water peak height changes (%) for the optogenetic stimulation during the paradigm. The shaded areas indicate periods of stimulation. **(D)** The unsuppressed water peaks were temporally averaged for the resting periods (black) and stimulated periods (green), demonstrating a difference in amplitude (arrow) that can be attributed to the BOLD effects. The averaged water peaks were used for further quantification of the metabolite concentrations.

For an improved characterization of the BOLD effects on the ^1^H-MR spectra, water, NAA, and tCr signals were measured over time during optogenetic stimulation paradigms (5 min ON-5 min OFF). The NAA peak height followed the optogenetic stimulation paradigm [Stimulation vs. REST vs. Stimulation: 8.4 ± 6% vs. 3.2 ± 4.3% (*p* > 0.05) vs. 4.5 ± 4.4%; *p* < 0.05; Paired Student's *t*-test; Figure 6A]. The relative change of the tCr peak height did not follow the paradigm of the stimulation (Figure 6B).

Unsuppressed water signals were also acquired during the paradigm of the stimulation as a function of time (*n* = 5) (Figure 6C).

Green laser stimulation induced significant increases in the water peak heights over 5 min of the S1FL activation relative to the rest periods (REST vs. Stimulation vs. REST vs. Stimulation: 0.1 ± 0.7% vs. 0.8 ± 1.2 % (*p* = 0.013) vs. −1.6 ± 1.4 % (*p* < 0.001) vs. 1.1± 1.5 (*p* < 0.001), Student's *t*-test; Figure 6C; mean ± standard deviation). The stimulated and resting water peaks were averaged into single water peaks, as shown overlaid in Figure 6D. The mean relative linewidth changes in water (Δlw_water_) were −0.5 ± 0.8%. The mean peak height and linewidth changes between REST and stimulation are summarized in Table 2.

**Table 2:** *In vivo* relative percent change of the height and linewidth of the water, NAA, and tCr peaks (optogenetic vs. REST).

**% Height change**			**% Linewidth change**		
**Water**	**NAA**	**tCr**	**Water**	**NAA**	**tCr**
1 ± 1.0	3.3 ± 7	NA	−0.5 ± 0.8	−2.8 ± 4	−1.4 ± 4

### 3.3 *In vivo* quantification of metabolite concentration changes affected by BOLD effects

**Figure 7:**
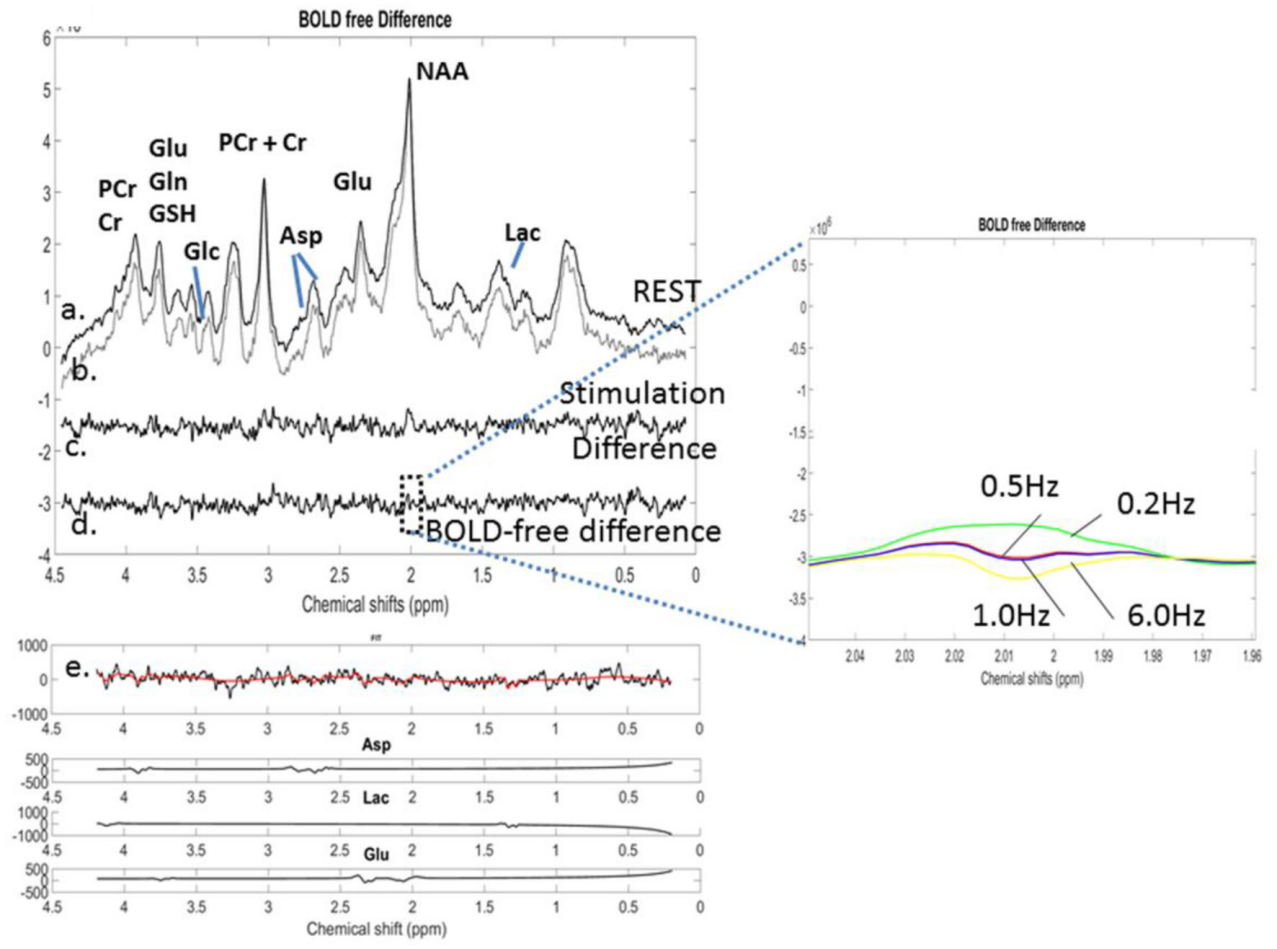
o-fMRS: Averaged (*n* = 8) and labeled proton MR spectra for **(a)** resting conditions and **(b)** optogenetic stimulation with green light. **(c)** The REST spectrum was subtracted from the stimulated spectrum before any correction for the BOLD effects. **(d)** The BOLD-free difference spectrum was obtained following the application of 0.5 Hz line broadening to minimize the BOLD effects. **(e)** LCModel fitting of the BOLD-free difference spectrum allowed the identification of the polarity of the metabolic changes and retrieved Asp, Lac, and Glu components included in the simulated basis set of the BOLD-free difference spectrum. Inset: NAA residuals for lb = 0.2 Hz, 0.5 Hz, 1 Hz, and 6 Hz resulting from the subtraction of the REST spectrum from the line-broadened stimulated spectrum. The NAA residual was best minimized for lb = 0.5 Hz.

The population-averaged ^1^H-MR spectra (*n* = 8) acquired during the optogenetic stimulation and REST are depicted in Figures 7a, b, respectively. The REST spectrum was subtracted from the stimulated spectrum, resulting in a difference spectrum (Figure 7c). The NAA peaks of the stimulated and REST spectra were line-matched using a line-broadening factor of 0.5 Hz. The lb factor was gradually increased in steps of 0.2 Hz up to 1 Hz by minimizing the residual NAA difference peak (Inset). Each ^1^H-MR spectrum was individually fitted using LCModel. Their subtraction resulted in a BOLD-corrected difference spectrum (Figure 7d). An identical line-matching procedure was performed using lb = 6 Hz, corresponding to a 6% change in the NAA peak height during the optogenetic stimulation. The 6 Hz line broadened spectrum was also fitted using LCModel. The metabolite concentrations quantified using LCModel and their changes are summarized in Table 3.

**Table 3:** Quantification of the *in vivo* metabolite concentrations: Baseline, stimulated, and BOLD-corrected metabolite concentrations were obtained through the adjustments of the MR spectra using LCModel.

	**Baseline concentration**		**Stimulated**								**BOLD corrected**							
	**REST**	**CRLB**	**No line width correction**				**STM/WaterBOLD**				**Line width matching ib** = **0.5 Hz**				**Line width matching ib** = **6 Hz**			
					**Concentration difference**				**Concentration difference**				**Concentration difference**				**Concentration difference**	
				**CRLB**	**Mean (**μ**mol/g)**	**Mean (%)**		**CRLB**	**Mean (**μ**mol/g)**	**Mean (%)**		**CRLB**	**Mean (**μ**mol/g)**	**Mean (%)**		**CRLB**	**Mean (**μ**mol/g)**	**Mean (%)**
Asp	4.31	10%	3.13	7%	−1.18	−27.3^***^	3.15	8%	−1.16	−26.8^***^	4.07	8%	−0.24	−5.5^**^	4.06	6%	−0.26	−5.9^**^
GIn	3.79	3%	3.58	3%	−0.21	−5.6^*^	3.53	3%	−0.26	−6.9^**^	3.58	4%	−0.21	−5.5^*^	3.37	3%	−0.41	−10.9^***^
GIn	6.98	2%	7.54	2%	0.56	8.05^***^	7.37	2%	0.40	5.66^***^	7.52	2%	0.54	7.7^***^	8.57	2%	1.59	22.8^***^
GSH	1.44	5%	1.20	6%	−0.23	−16.3^**^	1.17	7%	−0.27	−18.6^**^	1.51	5%	0.07	5.1	1.84	4%	0.40	28.0^***^
Ins	2.91	5%	3.64	3%	0.73	25.1^***^	3.82	4%	0.91	31.2^***^	3.54	6%	0.63	21.6^***^	3.58	3%	0.66	22.8^***^
NAA	8.70	2%	9.00	2%	0.30	3.5^***^	8.71	2%	0.01	0.14	8.47	2%	−0.23	−2.7	8.85	1%	0.15	1.76
Tau	4.44	3%	4.33	2%	−0.11	−2.5	4.26	3%	−0.18	−3.94	3.97	3%	−0.47	−10.5^***^	4.14	2%	−0.29	−6.62^***^
GIyc	2.05	5%	1.64	6%	−0.41	−19.9^***^	1.35	8%	−0.70	−34.3^***^	1.60	11%	−0.45	−21.8^***^	1.44	5%	−0.61	−29.84^***^
tNAA	9.44	2%	10.57	1%	1.12	11.9^***^	10.67	2%	1.22	12.95^*^	10.47	2%	1.02	10.8^***^	10.85	1%	1.41	14.91
tCr	8.00	2%	7.64	1%	−1.36	−4.5^**^8	7.52	2%	−0.48	−6.04^***^	8.18	2%	0.18	2.3	7.73	1%	−0.27	−3.40^**^
GIx	10.77	2%	11.12	2%	0.35	3.3^***^	10.90	2%	0.14	1.25	11.10	2%	0.33	3.1^***^	10.94	2%	0.18	1.64

The stimulated MR spectra were quantified using waterREST and waterBOLD. The optimized line-broadening factor (see Figure 7) was obtained for lb = 0.5 Hz. A non-data-driven lb = 6 Hz was also used. A group analysis was performed, and one-way ANOVAs were performed to evaluate the significance of the metabolite concentration changes. Bonferroni *post-hoc* tests were performed for conducting multiple comparisons. ^*^ *p* < 0.05; ^**^ *p* < 0.01; ^***^ *p* < 0.001.

Linewidth-matching techniques resulted in a reduction in the average metabolite concentrations by −0.06 ± μmol/g on average for lb = 0.5 Hz and −0.18 ± μmol/g for lb = 6 Hz compared to the uncorrected concentrations. The number of metabolite concentrations changing significantly due to stimulation differed with each analysis: waterBOLD vs. STIMC (lb = 0.5 Hz): 9 metabolites out of 11, *p* < 0.01; waterBOLD vs. STIMC (lb = 6 Hz): 4 metabolites out of 11, *p* < 0.01; and STIMC (lb = 0.5 Hz) vs. STIMC (lb = 6 Hz): 6 metabolites out of 11, *p* < 0.01.

Relative to STIM, water scaling with waterBOLD induced modest metabolic concentration changes (below ± 0.3 μmol/g), whereas the line-matching techniques induced important concentration changes for the Asp and Glu concentrations (approximately −1.0 μmol/g). The changes in the Glu concentrations were in the range of +0.4–+1.59 μmol/g for all BOLD corrections and remained highly significant (Table 3). This was also the case for the Asp concentrations, ranging from −1.16 to −0.24 μmol/g.

In a manner identical to the previous simulated data, the total BOLD effects measured *in vivo* can be calculated as the sum of the BOLD effects on water and metabolites. As for the simulated data, the BOLD effects on water were calculated as the difference between the metabolite concentrations quantified on the stimulated MR spectra using waterREST and waterBOLD. Assuming that line-broadening correction leads to BOLD-free metabolite concentrations, the BOLD effects on the metabolites were calculated as the difference between the stimulated metabolite concentrations scaled with waterBOLD and the BOLD-free metabolite concentrations. Thus, for NAA assumed as an intracellular metabolite and a neuronal marker:


$$\begin{array}{l} BOLD_{\text{G}}\left(NAA\right)=BOLD_{\text{water}}+BOLD_{\text{metab}}=3.3\%+2.91\%\\ \;=6.2\%and\text{Δ}\left[NAA\right]=-2.7\%\\ \;=0.29+0.24=0.53\text{ μ}mol/g \end{array}$$

For Glu, the same was applied:


$$\begin{array}{l} BOLD_{\text{G}}\left(Glu\right)=BOLD_{\text{water}}+BOLD_{\text{metab}}=2.3\%-1.93\%\\ \;=0.37\%and\text{Δ}\left[Glu\right]=7.7\%\\ \;=0.16-0.14=0.02\text{ μ}mol/g \end{array}$$

Glu represents a neuronal marker but is also present in the extracellular space during the synaptic release (Moussawi et al., 2011). Exchanges between intracellular and extracellular spaces may explain the negative effect on metabolites and the lesser effect on global BOLD (BOLD_G_). Similarly, BOLD effects on a glial marker, such as Ins, can be separated. The negative contribution of BOLD effects on water may be attributed to changes in the water dynamics within astrocytes (Borrachero-Conejo et al., 2020).


$$\begin{array}{l} BOLD_{\text{G}}\left(Ins\right)=BOLD_{\text{water}}+BOLD_{\text{metab}}=-4.7\%+7.85\%\\ \;=3.15\%and\text{Δ}\left[Ins\right]=\;21.6\%\\ \;=-0.18+0.28=0.1\text{ μ}mol/g \end{array}$$

BOLD effects can thus substantially contaminate metabolite concentrations.

## 4 Discussion

The present study examined the impact of BOLD effects and their correction on the quantification of metabolites using simulations and *in vivo* proton MR spectra acquired in the rat primary somatosensory cortex during optogenetic stimulation and rest periods. In rodents, the estimation of reliable metabolite concentration changes due to a functional challenge remains difficult and requires large animal populations (Sonnay et al., 2017) and higher magnetic fields or cryoprobes (Sonnay et al., 2017; Iordanova et al., 2015) to enhance the SNR.

### 4.1 The line-matching procedure removes BOLD effects

The removal of BOLD effects in ^1^H-fMRS allows for the estimation of metabolite concentration changes solely attributed to neurochemical changes as a consequence of brain activity (Moreno et al., 2013; Mangia et al., 2006, 2007b; Schaller et al., 2013; Bednarík et al., 2018; Schaller et al., 2014; Lin et al., 2012; Bednarík et al., 2015; Just et al., 2013; Just and Sonnay, 2017; Just, 2021). BOLD effects generate small changes in metabolite concentrations, which are not necessarily of identical magnitude for all metabolites (Schrantee et al., 2023; Iordanova et al., 2015). When not corrected, metabolic concentration changes may appear erroneously significant (Schaller et al., 2014). The appropriate line-broadening correction for BOLD effects is based on the value that best minimizes the residuals of tCr and NAA peaks resulting from the subtraction of population-averaged stimulated and REST proton MR spectra. This procedure strongly relies on an optimized phasing of both stimulated and REST spectra to limit potential frequency drifts during the subtraction. Low SNR levels in small VOIs of the rat cortex further complicate the subtraction methodology. A more direct quantification of BOLD-free metabolite concentration changes with LCmodel could simplify ^1^H-fMRS data analysis and interpretation.

Recent studies reported that LCModel analysis of short-TE data was highly sensitive to noise and spectral linewidth variations (Just, 2021; Zhang and Shen, 2020). Notably, the line broadening of the original data analyzed with LCModel induced substantial metabolite quantification changes. Therefore, the reliability of metabolite quantification changes following linewidth matching for BOLD correction can be questioned. In the present study, the simulation of the BOLD effects demonstrated that the line-matching procedure is an effective method to remove them with a quantification error of < 1% (±0.05 μmol/g). However, if the line-broadening factor is different from the line-narrowing factor induced by BOLD effects, up to a 5% (±0.3 μmol/g) error in quantification may be obtained, which is substantial when absolute metabolic changes as low as 0.2 μmol/g are expected (Mangia et al., 2007a,b; Schaller et al., 2013).

### 4.2 Water scaling

For the absolute quantification of metabolites using LCModel, the unsuppressed water peak acquired in the same VOI as the ^1^H-MR spectrum serves for scaling and eddy current correction. During stimulation, the water peak undergoes a line-narrowing effect due to BOLD effects. As LCModel (Provencher, 1993) considers resonance areas, the water line-narrowing due to ${\text{T}}_{2}^{*}$-induced effects does not affect metabolite quantification. The simulations used in the present study showed that this was not the case. The error induced on the metabolite concentrations was lower when waterBOLD was used for scaling compared to waterREST. The quantification of the metabolite concentrations on a proton MR spectrum with BOLD-simulated contamination suggested that compensatory effects take place when stimulated water (waterBOLD) is used. This finding led us to postulate that the metabolite concentrations quantified using waterBOLD are affected by BOLD effects on metabolites only, whereas the metabolite concentrations quantified using waterREST are affected by cumulative BOLD effects on metabolites and water. In single-voxel MR spectroscopy, lineshapes can be distorted by B0 field inhomogeneities and eddy currents. Metabolite fitting models using water reference lines to compensate for a B0 field inhomogeneity have also been used in combination with LCModel (Provencher, 1993) for quantification purposes. Notably, increased accuracy and stability of spectral quantification were found using the reference lineshapes from the water signal (Hong et al., 2019). In the present study, the use of waterBOLD for scaling partially corrected for the change in the spectral linewidth caused by BOLD effects.

### 4.3 Compartmentation of BOLD effects

BOLD responses measured using fMRI or fMRS on water represent ${\text{T}}_{2}^{*}$-induced effects encompassing both intravascular and extravascular compartments. Since BOLD fMRI provides an indirect measure of neuronal activity and relies on neurovascular coupling mechanisms, diffusion magnetic resonance imaging (dfMRI) (Le Bihan et al., 2006) has been proposed as a possible method for disentangling the vascular and extravascular components of BOLD. dfMRI relies on microstructural changes driven by neural activity, such as cell swelling, to induce changes in the diffusivity of water molecules.

Another technique independent of neurovascular coupling and taking advantage of the specific distribution of metabolites within different tissue compartments is MRS. Neuronal compartments and other extravascular and intravascular compartments may be differentially affected by ${\text{T}}_{2}^{*}$-induced effects. For example, ${\text{T}}_{2}^{*}$-induced effects measured using ^1^H-fMRS on the neuronal markers NAA and Glu and on the glial marker Ins may represent the effects on the neuronal compartments and the glial compartment, respectively (Zhu and Chen, 2001). In the human visual cortex at 4T, the changes in the linewidth and amplitude were similar for water and metabolites (Zhu and Chen, 2001), which was not the case in the present study with the human visual cortex at 9.4T. Thus, the linewidth and amplitude changes of the spectral lines assessed using ^1^H-fMRS could represent specific markers of BOLD effects in extravascular compartments.

Previous studies revealed that following injections of a paramagnetic agent (Dy-TTHA)^3−^ and a vasodilator, the water peak in the rat brain divided into three peaks, representing intracellular water, extravascular–extracellular water, and intravascular water. The distribution of Na^+^ ions in the three different compartments was also shown (Naritomi et al., 1987). When blocking the sodium pump with ouabain, a slight increase in the intracellular water peak signal was noticed, corresponding to an increase in the intracellular volume. The intravascular and extravascular–extracellular volumes became almost negligible.

By extrapolation, the BOLD effects assessed using MRS on a spectral water peak could be compared to the changes observed in a previous study (Naritomi et al., 1987). Moreover, upon depolarization induced by a stimulus, a Na^+^ gradient toward the intracellular milieu occurs. This analogy was used in the past with diffusion magnetic resonance imaging (dMRI) to verify that the dynamic swelling of neurons due to neuronal activation induced a decrease in the apparent diffusion coefficient (ADC) (Radecki et al., 2014). Potential changes in physical environments inducing ADC changes would also affect T_2_. Since T_2_ changes also occur as a result of focal cortical activation, cell swelling or macromolecular changes could also be envisaged in the present study. Other groups also demonstrated important BOLD effects on the neuronal compartment, whose origin was also attributed to cell swelling or macromolecular changes (Zhu and Chen, 2001; Lei et al., 2003). At 9.4T, a negligible effect of the intravascular BOLD effects was revealed (Lei et al., 2003). If cell swelling due to neural activation is assumed, then vascular BOLD effects can also be neglected. Changes in water and NAA signals during optogenetic stimulation could therefore be related to an increase in the intracellular volume. Diffusion functional magnetic resonance spectroscopy (dfMRS) was also proposed as a new tool for separating the changes in the properties of neuronal spaces from the hemodynamic response during neuronal activity (Branzoli et al., 2013). Despite the potential of both diffusion techniques, many measurements remain to be performed to ensure their validation as probes for the direct measurement of neural activity. In addition, both methods require additional work to validate the underlying mechanisms, and both are suspected to remain contaminated by residual BOLD signals. The present research contributed to the effort to better identify genuine neural activity. A simple method was proposed to separate water and intracellular BOLD effects, and this method will need to be validated.

### 4.3 *In vivo* evaluation of BOLD effects

The evaluation of the *in vivo* water and NAA peak amplitude changes as a function of time followed the stimulation paradigm during the periods of the optogenetic stimulation and the periods of REST, mimicking the changes in BOLD effects usually seen in BOLD fMRI time courses. These findings suggest that estimating water and NAA peak height changes is a reliable way to identify spectral BOLD effects in the rat cortex. Contrarily, the changes in the tCr peak amplitudes as a function of time did not follow the paradigm of stimulation. In a very recent study conducted at 9.4T in the human brain, tCr concentrations did not follow the visual stimulation paradigm, while Cr concentrations did (Dorst et al., 2022). These results confirm that BOLD effects may be counterbalanced by the conversion of PCr into Cr and vice versa, as hinted by the absence of observable T_2_ changes in other studies (Lei et al., 2003). In rodents, medetomidine sedation may also be responsible for some modulation of metabolite levels (Boretius et al., 2013).

*In vivo* stimulated spectra needed to be corrected for the BOLD effects without impacting the potential metabolite concentration changes due to optogenetic stimulation. Overall, the corrected amplitude of the changes in the metabolite concentrations upon stimulation, as well as the direction (positive or negative) of these changes, was in agreement with previous studies in rodents (Just et al., 2013; Just and Sonnay, 2017; Xu et al., 2005; Just and Faber, 2019; Sonnay et al., 2017) for all the different methods. The best agreement with previous literature values on functional metabolic concentration changes was found for the line-matching procedure (Just et al., 2013; Just and Sonnay, 2017; Xu et al., 2005; Sonnay et al., 2017) with lb = 0.5 Hz. Using the line-matching procedure with lb = 6 Hz, changes in the Asp and Glu concentrations were above −1 μmol/g (Table 3), which were largely overestimated compared to previous reports (Just et al., 2013; Just and Sonnay, 2017; Xu et al., 2005). Moreover, the linewidth-matching correction with lb = 6 Hz was demonstrated to be erroneous (Figure 7). Given these results, the line-matching procedure using lb = 0.5 Hz appeared to be the most suitable for correcting BOLD effects without affecting metabolic concentration changes due to optogenetic stimulation in the present study.

## 5 Conclusion

In conclusion, BOLD effects can be quantified using ^1^H-fMRS in the rat cortex. Linewidth-matching techniques remain the most reliable method for correcting false-positive errors in metabolite concentration estimates, provided the extent of ${\text{T}}_{2}^{*}$-induced effects is characterized to precisely determine the line-broadening factor. The ensuing calculation of a BOLD-corrected difference spectrum should then be more straightforward and specific. Alternatively, BOLD-related spectral changes may be useful for further identifying differences between cortical extravascular compartments upon activation. As pointed out in a recent study (Ligneul and Fernandes, 2021), novel ${\text{T}}_{2}^{*}$ methods are needed to better correct for BOLD effects in ^1^H-fMRS. At the same time, a better characterization of BOLD effects independent of neurovascular coupling may be useful for an improved assessment of direct neuronal activity.

## Data Availability

The original contributions presented in the study are included in the article/supplementary material, further inquiries can be directed to the corresponding author.
